# Multi-host environments select for host-generalist conjugative plasmids

**DOI:** 10.1186/s12862-016-0642-z

**Published:** 2016-04-02

**Authors:** Anastasia Kottara, James P. J. Hall, Ellie Harrison, Michael A. Brockhurst

**Affiliations:** Department of Biology, University of York, Wentworth Way, York, YO10 5DD UK

## Abstract

**Background:**

Conjugative plasmids play an important role in bacterial evolution by transferring ecologically important genes within and between species. A key limit on interspecific horizontal gene transfer is plasmid host range. Here, we experimentally test the effect of single and multi-host environments on the host-range evolution of a large conjugative mercury resistance plasmid, pQBR57. Specifically, pQBR57 was conjugated between strains of a single host species, either *P. fluorescens* or *P. putida*, or alternating between *P. fluorescens* and *P. putida.* Crucially, the bacterial hosts were not permitted to evolve allowing us to observe plasmid evolutionary responses in isolation.

**Results:**

In all treatments plasmids evolved higher conjugation rates over time. Plasmids evolved in single-host environments adapted to their host bacterial species becoming less costly, but in the case of *P. fluorescens*-adapted plasmids, became costlier in *P. putida*, suggesting an evolutionary trade-off. When evolved in the multi-host environment plasmids adapted to *P. fluorescens* without a higher cost in *P. putida*.

**Conclusion:**

Whereas evolution in a single-host environment selected for host-specialist plasmids due to a fitness trade-off, this trade-off could be circumvented in the multi-host environment, leading to the evolution of host-generalist plasmids.

## Background

Conjugative plasmids mediate genetic exchange in bacterial communities promoting bacterial adaptation and diversification [1]. Broad host range (BHR) conjugative plasmids, which can transmit between and be stably maintained across phylogenetically diverse hosts, play a particularly important role because they traffic ecologically important accessory genes between species [2, 3]. While broad host ranges benefit plasmids by increasing available hosts, evolutionary theory suggests that the evolution of ecological generalists, such as BHR plasmids, is likely to be constrained by fitness trade-offs [4–6]. Thus single-host environments are expected to select for specialist plasmids, whereas generalist plasmids are expected to evolve in environments where they regularly encounter multiple host bacterial species [7].

Previous studies have reported evolutionary changes in the effects of plasmid carriage across different host species following experimental evolution in single or multi-host environments [7–12]. Specifically, the BHR plasmid pB10 adapted to the originally unfavourable host *P. putida* H2 in a single-host environment [11], whereas in a multi-host environment, *Stenotrophomonas maltophilia* P21 and *P. putida* H2, adaptation of pB10 to either host species was impeded [7]. A key limitation of previous studies however is that they allow extended periods of bacterium-plasmid co-adaptation, which makes it difficult to disambiguate plasmid adaptation from host adaptation to understand how the plasmids themselves adapt to their hosts. To overcome this limitation here we held the bacterial hosts in evolutionary stasis while allowing only the plasmid to evolve by conjugating the evolving plasmid population into the ancestral bacterial host genotype(s) every 24 h. Specifically, to investigate the role of bacterial host species heterogeneity on plasmid evolution we experimentally evolved the environmental mercury resistance plasmid pQBR57 under single host, *Pseudomonas fluorescens* or *Pseudomonas putida*, or multi-host, both *P. fluorescens* and *P. putida*, treatments. We observed evidence for a fitness trade-off in plasmids adapted to the single-host *P. fluorescens* treatment, but that exposure to *P. putida* in the multi-host treatment allowed plasmids to circumvent this trade-off.

## Results and discussion

**Fig. 1:**
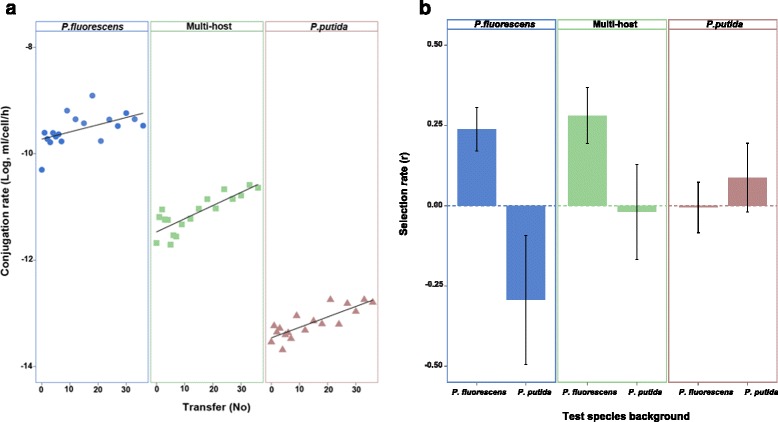
**a** Conjugation rate over time for plasmids in the single-host and multi-host treatments (Solid circle: Conjugation in *P. fluorescens*; Solid square: Conjugation between *P. fluorescens* and *P. putida*; Solid triangle: Conjugation in *P. putida*; Black line: linear regression); **b** Selection rate of *P. fluorescens* or *P.putida* carrying evolved plasmids from the single and multi-host treatments relative to isogenic strains carrying the ancestral plasmid. Selection rate of 0 indicates no difference between test and reference strains (dotted line), error bars: ±SEM)

The conjugation rate of pQBR57 varied between selection treatments (main effect of selection treatment, chi-square test, *Χ*^2^(2, *Ν* = 432) = 30.49, *p* = 2.39e-07), owing to a lower conjugation rate in *P. putida* than *P. fluorescens*, but increased over time in all treatments (main effect of time, chi-square test, *Χ*^2^(1, *Ν* = 432) = 18.24, *p* = 1.94e-05) (Fig. 1a). This suggests that pQBR57 adapted to the selection regimes by increasing its conjugation rate. In our experimental set-up, which involved both horizontal and vertical plasmid replication, conjugation is an essential part of the plasmid life-cycle; thus increasing conjugation rate is equivalent to increasing replication rate and therefore perhaps a predictable response to selection. However, increases in conjugation rate can be linked to increased costs of plasmid carriage [9, 13], which would impair the plasmid’s spread by vertical transmission (i.e. growth of transconjugants).

To estimate the fitness effects of carrying the evolved plasmids for host bacteria we competed bacteria carrying evolved plasmids against bacteria carrying the ancestral plasmid, in both host species backgrounds. The fitness effect of evolved plasmids depended on the combination of selection treatment and the test host species background (Fig. 1b; effect of species background and selection treatment interaction, factorial ANOVA, F_2,36_ = 4.50, *p* = 0.017). We observed that plasmids from the single-host *P. fluorescens* treatment evolved lower costs in *P. fluorescens*, but that this adaptation was accompanied by an increased cost in *P. putida* relative to the ancestral plasmid (Welch’s *t*-test, t_6.81_ = 2.592, *p* = 0.036) (Fig. 1b). Contrastingly, although plasmids from the single-host *P. putida* treatment evolved marginally lower costs in *P. putida*, we observed no change to the cost of carriage in *P. fluorescens* (Welch’s *t*-test, t_9.88_ = -0.618, *p* = 0.55) (Fig. 1b). Together this suggests an asymmetric trade-off, whereby pQBR57 adapted to *P. fluorescens* suffers a fitness trade-off in *P. putida*, but that there is not a corresponding fitness trade-off associated with adaptation to *P. putida*. Although we do not know the mechanism underlying the fitness trade-off in this study, previous work suggests that costs of plasmid carriage can arise from a range of mechanisms, including: the metabolic burden, expression of plasmid genes, copy number variation, and interference between plasmid and host cell regulatory systems [14, 15]. It is tempting to speculate that the last of these, regulatory interference, might be the most host-specific and thus more likely to generate the observed fitness trade-off [16].

Interestingly, evolved plasmids from the multi-host treatment evolved reduced cost-of-carriage in *P. fluorescens* but without increasing their cost-of-carriage in *P. putida* (Fig. 1b). This suggests that adaptation in a multi-host environment allowed pQBR57 to circumvent the fitness trade-off associated with adaptation to *P. fluorescens* in the single-host treatment. We do not know the specific mutations involved in plasmid adaptation in our experiment but the contrasting responses to selection between treatments suggests different genetic mechanisms. In particular, it seems likely that the different responses to selection in the *P. fluorescens* single-host treatment versus the multi-host treatment are due either to the fixation of different mutations, or the fixation of additional mutation(s) in the multi-host treatment to ameliorate the cost in *P. putida* of plasmid adaptation in *P. fluorescens*.

Environmental heterogeneity is thought to play a key role in the evolution of generalism and specialism in a wide variety of species [17]. Heterogeneous environments are predicted to select for generalist genotypes whereas homogeneous environments select for specialist genotypes [5]. For example, evolution experiments with algae adapting to light and dark show that algae adapted to light have lower fitness in dark environments and vice versa, whereas algae exposed to both environments evolve to be generalists [18]. We show that this evolutionary principle also applies to the evolution of mobile genetic elements in different hosts, in this case a conjugative plasmid. We provide evidence for a fitness trade-off associated with adaptation to a single host environment. The appearance of a fitness trade-off can be due, at the genetic level, to antagonistic pleiotropy or mutation accumulation [5, 18]. It seems more likely that the pattern observed here is the result of antagonistic pleiotropy, since there was equal opportunity for mutation accumulation in all treatments, but the trade-off was asymmetric affecting only the plasmids evolving in one of the species (*P. fluorescens*). Interestingly, exposure to both host species in the multi-host treatment did not constrain adaptation*.* This suggests that fitness trade-offs can be circumvented if plasmids are exposed to alternative hosts. Diverse bacterial communities are likely therefore to select for broad host range plasmids and consequently promote interspecific horizontal gene transfer, with implications for understanding the spread of important plasmid-borne traits like antibiotic resistance.

## Conclusion

Evolution in a single-host environment selected for host-specialist plasmids due to a fitness trade-off, but this trade-off could be circumvented in the multi-host environment, leading to the evolution of host-generalist plasmids.

## Methods

### Bacterial & plasmid strains

*P. fluorescens* SBW25 is a plasmid free soil bacterium isolated from sugar beets grown at a field site in Oxford [19, 20] whereas *P. putida* KT2440 is a soil bacterium derived from toluene-degrading *P. putida* strain mt-2 [21]. Both strains were chromosomally modified by directed insertion of an antibiotic marker gene coding the resistance in streptomycin (Sm) or gentamicin (Gm) by using the mini-Tn7 transposon system [22]. pQBR57 is a 307 kb conjugative mercury resistance plasmid isolated by mercury resistance selection from the bacterial population inhabiting the sugar beet rhizosphere and phytosphere of sugar beets [23]. Briefly, a marked *P. putida* UWC1 host was released and allowed to acquire plasmids from the natural bacterial community by conjugation. Plasmid-containing isolates were then recaptured by selecting for mercury resistance [23]. As the primary host of the plasmid was not recovered the plasmid’s host-range in nature is still unknown.

### Selection experiment

In each treatment, plasmid pQBR57 was forced to conjugated between either: differentially marked lines of *P. fluorescens* SBW25; differentially marked lines of *P. putida* KT2440; or between *P. fluorescens* SBW25 and *P. putida* KT2440. Each treatment consisted of 6 replication lines. Donor bacteria carrying the plasmid were incubated with the plasmid free, differentially marked recipient bacteria in King’s medium B (KB) for 24 h at 28 °C with shaking (170 rpm/min), after which time a sample of the mixture was diluted and spread on solid media that contained mercury (II) chloride (20 μM) and antibiotics (10 μg/ml gentamicin or 200 μg/ml streptomycin) to select for transconjugant colonies. Twenty-four hours later, 25 transconjugant colonies were selected randomly and used as donors to conjugate with overnight cultures of plasmid-free recipient bacteria revived from frozen stocks. The antibiotic resistances of the bacterial strains were used to ensure the conjugative transfer of the plasmid from one host to the other at each transfer step.

### Conjugation assay

Plasmid conjugation rate was measured through-out the selection experiment. Saturated cultures of plasmid free recipients and plasmid-carrying donors were mixed in 1:1 ratio, diluted 100-fold in fresh KB media and incubated at 28 °C for 24 h. Densities of donors and recipients at the start and end of conjugation were estimated by diluting and spreading on KB agar containing either 10 μg/ml gentamicin or 200 μg/ml streptomycin. The density of transconjugants following conjugation were estimated by plating onto KB agar containing 20 μM mercury (II) chloride plus antibiotics to select for transconjugants. Conjugation rate (γ) was calculated using the end-point method [24].

### Competitive fitness assay

Following 36 conjugative transfers one plasmid-containing bacterial clone from each population was used as a donor for conjugation into *P. fluorescens* and *P. putida* bacterial host backgrounds. Relative fitness was measured by mixing differentially labeled test (containing evolved plasmid) and reference (containing ancestral plasmid) in 1:1 ratio, diluted 100-fold and incubated at 28 °C for 24 h. Samples were plated on selective KB agar plates at the beginning and end of the competition and relative fitness was calculated as the selection rate (r) [25, 26]. To remove marker effects the selection rate of the test strain was normalized to the fitness of the focal marked strain carrying the ancestral plasmid when competed against the opposite marker labelled strain carrying the ancestral plasmid.

### Statistics

The statistical analysis was carried out using the software RStudio, version 3.1.0 [27]. We fitted a repeated measures mixed-effect linear model to the longitudinal data of the conjugation rate using the lme4 package [28] testing the effect of treatment and transfer number on conjugation rate, with ‘population’ as a random effect to account for repeated measures. We used a linear model to analyse normalized selection rate of bacteria carrying the evolved plasmids, fitting test species, treatment, and their interactions as fixed effects. Welch’s *t*-test was used to compare selection rate between the test species within each treatment.
